# A Temporal Examination of Cytoplasmic Ca^2 +^ Levels, Sarcoplasmic Reticulum Ca^2 +^ Levels, and Ca^2 +^ -Handling-Related Proteins in Different Skeletal Muscles of Hibernating Daurian Ground Squirrels

**DOI:** 10.3389/fphys.2020.562080

**Published:** 2020-10-21

**Authors:** Zhe Wang, Jie Zhang, Xiu-Feng Ma, Hui Chang, Xin Peng, Shen-Hui Xu, Hui-Ping Wang, Yun-Fang Gao

**Affiliations:** ^1^Shaanxi Key Laboratory for Animal Conservation, College of Life Sciences, Northwest University, Xi’an, China; ^2^Key Laboratory of Resource Biology and Biotechnology in Western China, Northwest University, Ministry of Education, Xi’an, China; ^3^College of Life Sciences, Qufu Normal University, Qufu, China

**Keywords:** hibernation, calcium homeostasis, skeletal muscle, calcium pump, ryanodine receptor

## Abstract

To explore the possible mechanism of the sarcoplasmic reticulum (SR) in the maintenance of cytoplasmic calcium (Ca^2+^) homeostasis, we studied changes in cytoplasmic Ca^2+^, SR Ca^2+^, and Ca^2+^-handling proteins of slow-twitch muscle (soleus, SOL), fast-twitch muscle (extensor digitorum longus, EDL), and mixed muscle (gastrocnemius, GAS) in different stages in hibernating Daurian ground squirrels (*Spermophilus dauricus*). Results showed that the level of cytoplasmic Ca^2+^ increased and SR Ca^2+^ decreased in skeletal muscle fiber during late torpor (LT) and inter-bout arousal (IBA), but both returned to summer active levels when the animals aroused from and re-entered into torpor (early torpor, ET), suggesting that intracellular Ca^2+^ is dynamic during hibernation. The protein expression of ryanodine receptor1 (RyR1) increased in the LT, IBA, and ET groups, whereas the co-localization of calsequestrin1 (CSQ1) and RyR1 in GAS muscle decreased in the LT and ET groups, which may increase the possibility of RyR1 channel-mediated Ca^2+^ release. Furthermore, calcium pump (SR Ca^2+^-ATPase 1, SERCA1) protein expression increased in the LT, IBA, and ET groups, and the signaling pathway-related factors of SERCA activity [i.e., β-adrenergic receptor2 protein expression (in GAS), phosphorylation levels of phospholamban (in GAS), and calmodulin kinase2 (in SOL)] all increased, suggesting that these factors may be involved in the up-regulation of SERCA1 activity in different groups. The increased protein expression of Ca^2+^-binding proteins CSQ1 and calmodulin (CaM) indicated that intracellular free Ca^2+^-binding ability also increased in the LT, IBA, ET, and POST groups. In brief, changes in cytoplasmic and SR Ca^2+^ concentrations, SR RyR1 and SERCA1 protein expression levels, and major RyR1 and SERCA1 signaling pathway-related factors were unexpectedly active in the torpor stage when metabolic functions were highly inhibited.

## Introduction

Calcium (Ca^2+^) is homeostatically controlled in mammals (Thomas et al., 1996). Prolonged skeletal muscle disuse (e.g., during spaceflight, hindlimb unloading, and bed rest) can lead to disturbance of intracellular Ca^2+^ homeostasis, mainly exhibited by cytoplasmic Ca^2+^ overload (Ingalls et al., 2001; Wu et al., 2012; Hu et al., 2017). Cytoplasmic Ca^2+^ overload can activate the calpain protein degradation system and promote skeletal muscle protein degradation, which is an important mechanism leading to skeletal muscle atrophy (Goll et al., 2003; Gao et al., 2018).

Hibernation is an important strategy for survival under low environmental temperatures and food scarcity during the winter months (Martin and Yoder, 2014; van Breukelen and Martin, 2015; Nordeen and Martin, 2019). Numerous hibernators, including Daurian ground squirrels (*Spermophilus dauricus*), avoid loss of muscle mass and force during prolonged fasting and torpor inactivity, thus providing a natural model to study the mechanisms involved in the prevention of and resistance to disuse-induced skeletal muscle atrophy (Gao et al., 2012; Hindle et al., 2015). Our previous study showed that cytoplasmic Ca^2+^ is markedly elevated in the skeletal muscle fibers of ground squirrels during inter-bout arousal, but also shows partial recovery after inter-bout arousal, thus suggesting that intracellular Ca^2+^ is dynamic during different stages in hibernating ground squirrels (Fu et al., 2016). Periodic torpor-arousal cycles may be involved in the antagonism of skeletal muscle atrophy by alleviating excessive Ca^2+^ in the cytoplasm of muscle fibers and mitigating increased protein degradation. Therefore, studies on the potential mechanisms involved in Ca^2+^ homeostasis in the skeletal muscle fibers of hibernators are of great significance for revealing the mechanism controlling disuse-induced skeletal muscle atrophy.

A dynamic balance between intracellular pools and cytoplasmic Ca^2+^ is the main factor affecting intracellular Ca^2+^ homeostasis. The measurement of Ca^2+^ levels can thus reflect the maintenance or loss of intracellular Ca^2+^ homeostasis. In skeletal muscle, the endoplasmic reticulum is specialized into the sarcoplasmic reticulum (SR), an important organelle in the maintenance of intracellular Ca^2+^ homeostasis (MacIntosh et al., 2012). Research on Ca^2+^ homeostasis in skeletal muscles under disuse conditions has mainly focused on the SR, with changes in key Ca^2+^ handling proteins in the SR found to be closely related to cytoplasmic Ca^2+^ overload (Kraner et al., 2011).

Earlier studies have demonstrated that the increase in protein expression of the SR Ca^2+^ release channel ryanodine receptor1 (RyR1) and decrease in protein expression or activity of the calcium pump (sarco/endoplasmic reticulum Ca^2+^ ATPase isoform 1, SERCA1) are important mechanisms leading to Ca^2+^ overload and skeletal muscle atrophy in non-hibernating animals (Hunter et al., 2001; Donoghue et al., 2004; Kraner et al., 2011). Compared with non-hibernators (Silvani et al., 2018), only a few studies have been conducted on key SR Ca^2+^-handling proteins in hibernators, and results have been relatively inconsistent. For example, the protein expression levels of SERCA1, RyR1, and calsequestrin1 (CSQ1) are reported to decrease in the hindlimb skeletal muscles in Siberian ground squirrels (*Spermophilus undulatus*) during torpor (Malysheva et al., 2001). Conversely, our previous study showed that SERCA activity increases in the soleus (SOL) and extensor digitorum longus (EDL) muscles of ground squirrels during inter-bout arousal and torpor compared with that during pre-hibernation (Guo et al., 2017). Furthermore, calmodulin (CaM) protein expression is reported to increase in the mixed hindlimb skeletal muscles of thirteen-lined ground squirrels (*Ictidomys tridecemlineatus*) during torpor (Zhang and Storey, 2016b). Importantly, however, the underlying mechanisms related to the above changes, especially the specific regulation of RyR1 and SERCA activity, are still not clear.

We also previously found that distinct skeletal muscles respond to cytoplasmic Ca^2+^ overload after arousal from and re-entry into torpor in different ways (Fu et al., 2016). We hypothesize that maintaining the dynamic balance between SR and cytoplasmic Ca^2+^ through regulating SR Ca^2+^ uptake and excretion channels is an important mechanism used to avoid or reduce cytoplasmic Ca^2+^ overload in hibernators, and may be related to skeletal muscle type. To test this hypothesis, we used the SOL, EDL, and gastrocnemius (GAS) muscles of Daurian ground squirrels to study cytoplasmic and SR Ca^2+^ concentrations in skeletal muscle fibers, as well as changes in the expression levels of RyR1, SERCA1, Ca^2+^ binding proteins, and signaling pathway-related proteins to investigate the relationship between changes in SR Ca^2+^ and cytoplasmic Ca^2+^ levels and the expression of key Ca^2+^ handling proteins in skeletal muscles during different stages in hibernating ground squirrels.

## Materials and Methods

### Animals and Groups

**FIGURE 1:**
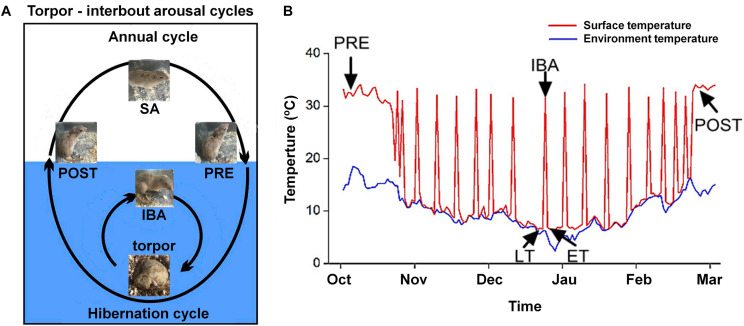
Diagram of hibernation cycle and body surface temperature in ground squirrels. **(A)** Animal state of annual and hibernation (blue shading) cycles in ground squirrels. **(B)** Body surface temperature of Daurian ground squirrels (red line) and environmental temperature (blue line) during hibernation. SA, summer active; PRE, pre-hibernation; LT, late torpor; IBA, inter-bout arousal; ET, early torpor; POST, post-hibernation.

All animal procedures and care and handling protocols were approved by the Committee on the Ethics of Animal Experiments of Northwest University (Permit Number: SYXK 2010-004). Daurian ground squirrels were caught within the Weinan region in Shaanxi Province, China. After transfer to our laboratory, the ground squirrels were individually housed in 50 cm × 50 cm × 20 cm cages. Animals were weight-matched and divided into six groups (male to female ratio of ∼1:2): (1) summer active (SA): samples were collected in mid-June from ground squirrels with a body surface temperature (Tb) range of 36–38°C; (2) pre-hibernation (PRE): samples were collected in mid-September from ground squirrels with a Tb range of 36–38°C; (3) late torpor (LT): samples were collected 2 months after first entering torpor from ground squirrels with a Tb range of 5–8°C for more than 5 days (d); (4) inter-bout arousal (IBA): samples were collected 2 months after first entering torpor from ground squirrels with a Tb range of 34–37°C for less than 12 h (h); (5) early torpor (ET): samples were collected 2 months after first entering torpor and after animals entered a new torpor bout with a Tb range of 5–8°C for less than 24 h; (6) post-hibernation (POST): samples were collected in March of the following year from ground squirrels aroused from torpor with a Tb range of 36–38°C for more than 2-day. The body surface and environmental temperatures are shown in Figure 1. As described in previous study, after the animals were anaesthetized with sodium pentobarbital (90 mg/kg), the skeletal muscles (slow-twitch SOL, fast-twitch EDL, and mixed GAS) were separately used for all experiments (Fu et al., 2016). Animals in each group were divided into two batches (*n* = 8 for each batch). The first batch was used for cytoplasmic and SR Ca^2+^ level measurement. The second batch was used for molecular biology experiments, including protein extraction, western blot analysis, and protein co-localization analysis. At the end of surgical intervention, the animals were sacrificed by an overdose injection of sodium pentobarbital.

### Cytoplasmic and SR Ca^2+^ Level Measurement

Muscle samples with tendons were dissected carefully from surrounding tissues and sarcolemma. The same volume of complete muscle from each type of skeletal muscle was separated along the longitudinal axis using tweezers (30-s process), then rinsed with 20 mL of phosphate-buffered saline (PBS, 137 mM sodium chloride, 4.3 mM disodium chloride, 2.7 mM potassium chloride, 1.4 mM monopotassium phosphate, pH 7.4), acutely dissociated with 3 mL of enzymatic digestion solution consisting of 0.35% collagenase I and 0.17% neutral protease (Sigma-Aldrich, Saint Quentin Fallavier, France), and finally incubated at 37°C on an orbital shaker for 2 h. The enzymatic digestion solution was saturated with 95% O_2_ and 5% CO_2_ gas mixture to ensure the muscle fibers were completely digested, after which the solution was removed with PBS and the muscles were agitated gently and repeatedly with pipettes (Wang et al., 2019). The dissociated single muscle fibers were set onto culture chamber slides and finally observed under an inverted microscope (Olympus, IX2-ILL100, Japan) (10-min process). One muscle fiber was separated on each slide in the incubator to avoid interaction of multiple muscle fibers during staining.

We used fluo-3-acetoxymethylester (Fluo-3/AM) (Invitrogen, Carlsbad, CA, United States), which demonstrates increased fluorescence upon Ca^2+^ binding, to determine cytoplasmic free Ca^2+^. The experimental method is the same as that in our previous report (Zhang et al., 2019). Briefly, after washing the samples three times with fresh PBS, dye (5 mM Fluo-3/AM) was slowly added along the sides of the single muscle fibers, followed by incubation in the dark at 37°C for 30 min. After incubation, the glass slide-mounted Fluo-3/AM-loaded fibers were washed with fresh PBS three times (20 s/time, 1-min process). The slide was quickly placed on the microscope stage, with the fibers focused in the bright field (20-s process) and scanned via laser confocal microscopy in combination with an Olympus FV10-ASW system (Tokyo, Japan) under 488-nm krypton/argon laser illumination, with fluorescence detected at 526 nm. According to their length, three to five pictures were captured at 10 × objective magnification for each muscle fiber (10-s capture process for each picture). Six different areas were randomly selected for fluorescence intensity measurements in each image. Total fluorescence intensity/total area of the selected region was used as the average fluorescence intensity of the muscle fiber, which represented the relative concentration of Ca^2+^. The average value of the measured result was taken as the fluorescence intensity of the muscle fiber cytosolic Ca^2+^ concentration. The average value of 10 muscle fibers was taken as the fluorescence intensity of the muscle fiber cytosolic Ca^2+^ concentration. Quantification analysis of the fluorescence intensity was performed with Image-Pro Plus 6.0.

Magnesium-Fluo-4-acetoxymethylester (mag-Fluo-4/AM) (M14206, Thermo Fisher Scientific, Rockford, IL, United States), which exhibits an increase in fluorescence upon binding to Ca^2+^, was used to indicate SR free Ca^2+^, as described previously (Park et al., 2000). Briefly, after washing samples twice with fresh PBS, dye (5 mM mag-Fluo-4/AM) was slowly added along the sides of the single muscle fibers, followed by incubation in the dark at 37°C for 30 min. After incubation, the glass slide-mounted mag-Fluo-4/AM-loaded fibers were washed with fresh PBS three times (20 s/time, 1-min process). The slide was then quickly placed on the microscope stage, with the fibers focused in the bright field (20-s process) and scanned via laser confocal microscopy in combination with an Olympus FV10-ASW system (Japan) under 488-nm krypton/argon laser illumination, with fluorescence detected at 526 nm. Analysis and statistical methods were similar to those used for the measurement of cytoplasmic Ca^2+^ mentioned above.

### Western Blot

A Nuclear/Cytosol Fractionation Kit was used to extract cytoplasmic protein (Biovision, #K266-25, Mountain View, CA, United States). As described previously (Zhang et al., 2019; Wang et al., 2020b), soluble protein concentrations were then detected using a Pierce^TM^ BCA Protein Quantification kit (Thermo Fisher Scientific, 23227, United States). The supernatants were mixed with 1 × SDS loading buffer (100 mM Tris, 5% glycerol, 5% 2-β-mercaptoethanol, 4% SDS, and bromophenol blue, pH 6.8) at a 1:4 v/v ratio, followed by boiling and then storage at −20°C for further analysis.

The muscle protein extracts were then separated via SDS-PAGE [10% Laemmli gel with an acrylamide/bisacrylamide ratio of 29:1 and 98% 2,2,2-trichloroethanol (Aladdin, JI522028, China)]. After electrophoresis, the proteins were electrically transferred to polyvinylidene fluoride (PVDF) membranes (0.45-μm pore size) using a Bio-Rad semi-dry transfer apparatus. The blotted membranes were blocked with 1% BSA in Tris-buffered saline (TBS; 150 mM NaCl, 50 mM Tris-HCl, pH 7.5) and incubated with primary antibodies in TBS containing 0.1% BSA at 4°C overnight. The primary antibodies used for western blot analysis are listed in Table 1. The membranes were then incubated with Goat anti-Rabbit IgG (H + L) Secondary Antibody (1:5000, 31460, Thermo Fisher, United States) or Goat anti-Mouse IgG (H + L) Secondary Antibody (1:5000, 62-6520, Thermo Fisher, United States) for 90 min at room temperature, covered with West Pico Plus Chemiluminescent Substrate (34580, Thermo Fisher, United States), and visualized with an scanner (G: box, GBOX Cambridge, United Kingdom). Quantification analysis of the blots was performed using NIH Image J software. Immunoblot band density in each lane was standardized against the summed densities of total protein.

**TABLE 1:** Primary and secondary antibodies used in western blot analysis.

Protein name	Antibody details
RyR1	1:1000, 8153S, CST, United States
DHPR	1:1000, SC-21781, Santa Cruz, United States
FKBP12	1:1000, ab58072, Abcam, United Kingdom
SERCA1	1:1000, 4219S, CST, United States
SLN	1:200, 18395-1-AP, Proteintech, China
PLB	1:1000, 8495S, CST, United States
P-PLB	1:1000, 8496S, CST, United States
β-AR2	1:500, ab182136, Abcam, United Kingdom
CaMK2	1:500, 3357S, CST, United States
P-CaMK2	1:1000, 12716S, CST, United States
CaM	1:1000, 4830S, CST, United States
CSQ1	1:10000, ab191564, Abcam, United Kingdom

### Protein Co-localization Analysis

We cut 10-μm thick frozen muscle cross-sections from the mid-belly of each muscle at −20°C with a cryostat (Leica, Wetzlar, CM1850, Germany), followed by storage at −80°C for further staining (Wang et al., 2020a). Immunofluorescence was used to determine co-localization with dihydropyridine receptor (DHPR)/RyR1, CSQ1/RyR1, 12-kDa FK506 binding protein (FKBP12)/RyR1, and sarcolipin (SLN)/SERCA1. After air drying for 2 h, the sections were incubated in a blocking solution (5% BSA) (Boster, Wuhan, China) for 10 min at room temperature and then incubated in a primary antibody solution at 4°C overnight. The following day, the sections were incubated with secondary antibody at 37°C for 2 h. After this, the sections were incubated with another primary and secondary antibody under the same conditions. The primary and secondary antibodies are listed in Table 2. Finally, the glass slides were placed in 4′-6′-diamidino-2-phenylindole (DAPI) (1:100, D9542, Sigma-Aldrich, United States) at 37°C for 30 min. Images were visualized using a confocal laser scanning microscope by krypton/argon laser illumination at 350, 488, and 647 nm emitted light, and captured at 461, 526, and 665 nm. Six figures were analyzed in each sample and eight samples were analyzed in each group. The co-localization of two proteins was calculated by Pearson’s correlation coefficients using Image-Pro Plus 6.0 (Lagache et al., 2015).

**TABLE 2:** Primary and secondary antibodies used in protein co-localization.

Protein name	Primary antibody details	Secondary antibody details
DHPR/RyR1	Rabbit anti-RyR1 (1:200, 8153S, CST, United States)	Goat anti-rabbit Alexa Fluor 647 (1:200, A21245, Thermo Fisher Scientific, United States)
	Mouse anti-DHPR (1:200, SC-21781, Santa Cruz, United States)	Goat anti-mouse FITC (1:200, F1010, Sigma-Aldrich, France)
CSQ1/RyR1	Rabbit anti-RyR1	Goat anti-rabbit Alexa Fluor 647
	Rabbit anti-calsequestrin 1 (1:100, ab191564, Abcam, United Kingdom)	Goat anti-rabbit Alexa Fluor 488 (1:200, 11034, Thermo Fisher Scientific, United States)
FKBP12/RyR1	Rabbit anti-RyR1	Goat anti-rabbit Alexa Fluor 647
	Mouse anti-FKBP12 (1:200, ab58072, Abcam, United Kingdom)	Goat anti-mouse FITC
SLN/SERCA1	Rabbit anti-SERCA1 (1:200, 4219S, CST, United States)	Goat anti-rabbit Alexa Fluor 647
	Rabbit anti-SLN (1:200, 18395-1-AP, Proteintech, China)	Goat anti-rabbit Alexa Fluor 488

### Statistical Analyses

The normality of data and homogeneity of variance were tested by Levene tests. Single factor analysis of variance (one-way ANOVA) was used to determine group differences. The Tukey *post hoc* test was used for multiple comparisons among groups. Differences were considered significant at *P* < 0.01 because the Daurian ground squirrels were wild captured, and thus individual differences were larger. Data in the tables are expressed as means ± standard deviation (means ± SD); data in the figures are expressed as individual sample values, and median, average, upper and lower quartiles, upper and lower edges, and extreme outliers are reported. All statistical analyses were conducted using SPSS 19.0.

## Results

### Body Weight, Skeletal Muscle Wet Weight (MWW), and Ratio of Skeletal Muscle Wet Weight to Body Weight (MWW/BW)

Compared with the body weights in the PRE group, the body weights during the experiment were 29–32% lower in the LT, IBA, ET, and POST groups. Compared with that in the SA group, the MWW showed no change in the SOL and EDL but a 22–25% decrease in the GAS in the LT, IBA, and ET groups. Moreover, compared with that in the PRE group, the skeletal muscle MWW/BW ratios were much higher in the other groups (*P* < 0.01). Thus, only slight skeletal muscle atrophy occurred in the different measured stages in hibernating ground squirrels (Table 3).

**TABLE 3:** Effects of hibernation on body weight (BW), muscle wet weight (MWW), and ratio of MWW/BW in Daurian ground squirrels.

			MWW during experiment (mg)			MWW/BW during experiment (mg/g)		
	BW before	BW during						
Group	experiment (g)	experiment (g)	SOL	EDL	GAS	SOL	EDL	GAS
SA	325.24 ± 27.46	325.24 ± 27.46^*a*^	131 ± 18	140 ± 18	257 ± 31^*a*^	0.40 ± 0.05^*a*^	0.43 ± 0.04^*bc*^	0.79 ± 0.10^*b*^
PRE	315.44 ± 21.82	315.44 ± 21.82^*a*^	101 ± 23	123 ± 27	261 ± 41^*a*^	0.32 ± 0.03^*b*^	0.39 ± 0.05^*c*^	0.82 ± 0.09^*b*^
LT	322.71 ± 18.49	218.71 ± 30.4^*b*^	99 ± 19	110 ± 32	211 ± 27^*ab*^	0.45 ± 0.05^*a*^	0.50 ± 0.06^*ab*^	0.96 ± 0.12^*a*^
IBA	330.14 ± 26.59	232.71 ± 32.07^*b*^	113 ± 24	118 ± 22	225 ± 28^*ab*^	0.49 ± 0.04^*a*^	0.51 ± 0.07^*ab*^	0.97 ± 0.11^*a*^
ET	319.51 ± 24.90	217.83 ± 25.70^*b*^	102 ± 18	114 ± 19	195 ± 32^*b*^	0.47 ± 0.03^*a*^	0.53 ± 0.04^*a*^	0.90 ± 0.08^*b*^
POST	319.39 ± 30.15	223.20 ± 31.43^*b*^	101 ± 15	112 ± 23	200 ± 34^*ab*^	0.45 ± 0.06^*a*^	0.50 ± 0.04^*ab*^	0.90 ± 0.08^*b*^

*SOL, soleus; GAS, gastrocnemius; EDL, extensor digitorum longus; SA, summer active; PRE, pre-hibernation; LT, late torpor; IBA, inter-bout arousal; ET, early torpor; POST, post-hibernation. Data represent means ± SD; n = 16. Different letters (such as a and b) indicate differences between period groups (P < 0.01), same letters (including a and ab) indicate no differences between period groups, and no letters indicate no differences among all six period groups.*

### Cytoplasmic Ca^2+^ Level in Single Muscle Fibers

**FIGURE 2:**
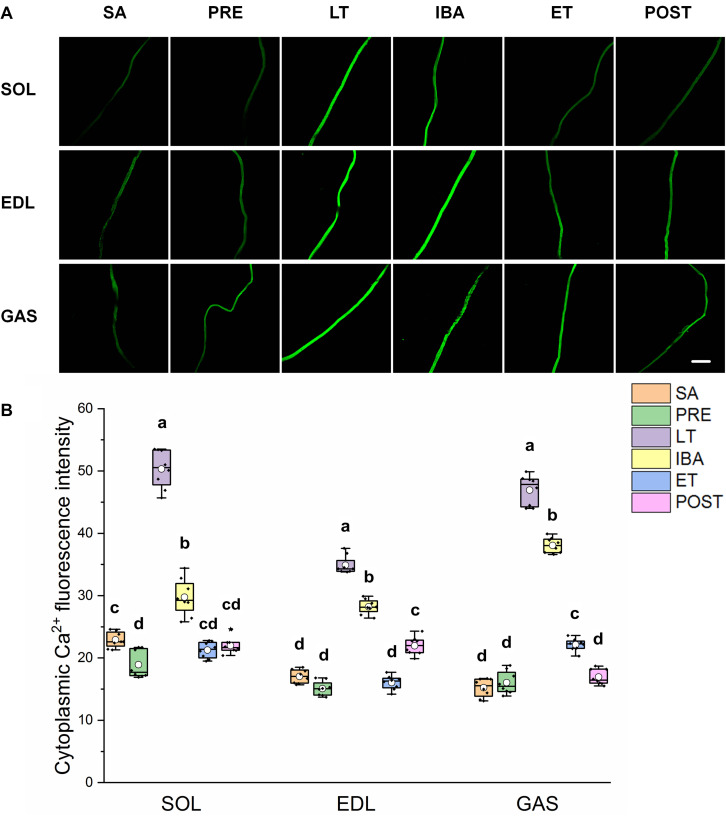
Changes in cytoplasmic Ca^2+^ fluorescence intensity of single muscle fiber in skeletal muscles during different periods. **(A)** Representative fluorescence images of single muscle fiber loaded by Fluo-3/AM. Scale bar = 200 μm. **(B)** Box-plot depicting changes in mean intensity of cytoplasmic Ca^2+^ fluorescence. Boxes represent upper and lower quartiles, middle horizontal line represents median, hollow circle represents average, lines extending from upper and lower ends represent upper and lower edges, respectively, asterisks represent extreme outliers, and points represent individual sample values. *n* = 8. SOL, soleus muscle; EDL, extensor digitorum longus; GAS, gastrocnemius muscle; SA, summer active group; PRE, pre-hibernation group; LT, late torpor group; IBA, inter-bout arousal group; ET, early torpor group; POST, post-hibernation group. Different letters (such as a and b) indicate differences between period groups (*P* < 0.01), same letters (including a and ab) indicate no differences between period groups, and no letters indicate no differences among all six period groups.

As shown in Figure 2, the same Ca^2+^ dynamics were observed in all three skeletal muscles. Compared with that in the SA and PRE groups, cytoplasmic Ca^2+^ was markedly elevated by 30–208% in the LT and IBA groups. During the torpor-arousal cycle, cytoplasmic Ca^2+^ decreased by 19–58% in the IBA and ET groups compared with that in the LT group. In addition, the levels were much lower in the ET group than that in the IBA group (*P* < 0.01). Overall, cytoplasmic Ca^2+^ was elevated to varying degrees in the different measured stages in hibernating ground squirrels (especially in the LT and IBA groups), but partially recovered when the animals aroused from and re-entered into torpor.

### SR Ca^2+^ Level in Single Muscle Fibers

**FIGURE 3:**
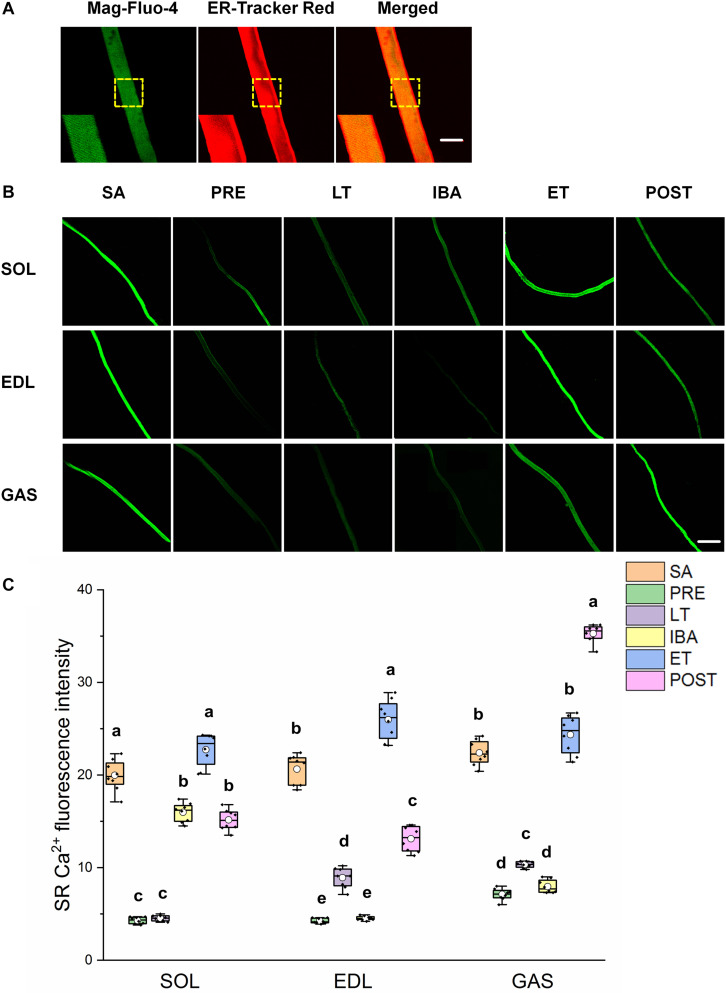
Changes in sarcoplasmic reticulum (SR) Ca^2+^ fluorescence intensity of single muscle fibers in skeletal muscles during different periods. **(A)** Representative fluorescence images of single muscle fibers loaded by mag-Fluo-4/AM and SR-Tracker Red dye. Mag-Fluo-4/AM (green) for SR Ca^2+^, SR-Tracker Red dye (red) for SR. Scale bar = 50 μm. **(B)** Representative fluorescence images of fluorescent SR Ca^2+^ in skeletal muscles during different periods. Scale bar = 200 μm. **(C)** Box-plot depicting changes in mean intensity of SR Ca^2+^ fluorescence. Boxes represent upper and lower quartiles, middle horizontal line represents median, hollow circle represents average, lines extending from upper and lower ends represent upper and lower edges, respectively, asterisks represent extreme outliers, and points represent individual sample values. *n* = 8. SOL, soleus muscle; EDL, extensor digitorum longus; GAS, gastrocnemius muscle; SA, summer active group; PRE, pre-hibernation group; LT, late torpor group; IBA, inter-bout arousal group; ET, early torpor group; POST, post-hibernation group. Different letters (such as a and b) indicate differences between period groups (*P* < 0.01), same letters (including a and ab) indicate no differences between period groups, and no letters indicate no differences among all six period groups.

As shown in Figure 3, similar alternations in the SR Ca^2+^ levels were observed in the three different skeletal muscles. Compared with that in the SA group, SR Ca^2+^ levels in the SOL, EDL, and GAS muscles decreased by 20–80% in the PRE, LT, and IBA groups. Compared with that in the LT group, SR Ca^2+^ increased by 136–401% in the ET and POST groups. In addition, SR Ca^2+^ was 42–472% higher in the ET group relative to the IBA group (*P* < 0.01). Overall, SR Ca^2+^ decreased during torpor and recovered when animals aroused from and re-entered into torpor, opposite to the pattern observed for cytoplasmic Ca^2+^.

### Relative Protein Expression Involved in RyR1

**FIGURE 4:**
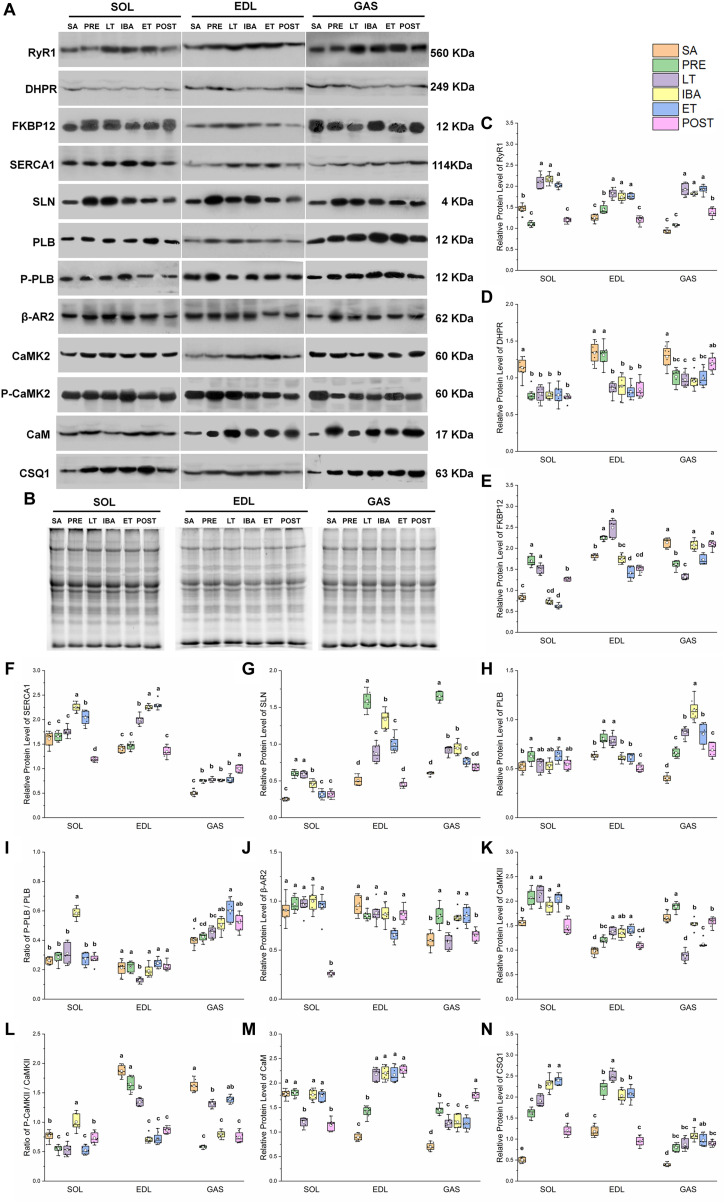
Changes in protein levels of RyR1, DHPR, FKBP12, SERCA1, SLN, PLB, β-AR2, CaMK2, CaM, CSQ1, ratios of P-PLB/PLB and P-CaMK2/CaMK2 in skeletal muscles during different periods. **(A)** Representative immunoblots of RyR1, DHPR, FKBP12, SERCA1, SLN, P-PLB, PLB, β-AR2, P-CaMK2, CaMK2, CaM, and CSQ1 in three different types of skeletal muscles during different periods. **(B)** Representative polyacrylamide gel of total protein. **(C)** Relative protein expression of RyR1. **(D)** Relative protein expression of DHPR. **(E)** Relative protein expression of FKBP12. **(F)** Relative protein expression of SERCA1. **(G)** Relative protein expression of SLN. **(H)** Relative protein expression of PLB. **(I)** Ratio of P-PLB to PLB. **(J)** Relative protein expression of β-AR2. **(K)** Relative protein expression of CaMK2. **(L)** Ratio of P-CaMK2 to CaMK2. **(M)** Relative protein expression of CaM. **(N)** Relative protein expression of CSQ1. Boxes represent upper and lower quartiles, middle horizontal line represents median, hollow circle represents average, lines extending from upper and lower ends represent upper and lower edges, respectively, asterisks represent extreme outliers, and points represent individual sample values. *n* = 8. SOL, soleus muscle; EDL, extensor digitorum longus; GAS, gastrocnemius muscle; SA, summer active group; PRE, pre-hibernation group; LT, late torpor group; IBA, inter-bout arousal group; ET, early torpor group; POST, post-hibernation group. Different letters (such as a and b) indicate differences between period groups (*P* < 0.01), same letters (including a and ab) indicate no differences between period groups, and no letters indicate no differences among all six period groups.

A similar pattern of change in RyR1 protein expression in the different groups was observed in the SOL, EDL, and GAS muscles (Figures 4A,B). Compared with that in the SA and PRE groups, RyR1 protein expression increased by 20–107% in the LT, IBA, and ET groups, but then recovered in the POST group (Figure 4C). The DHPR protein expression levels in the SOL, EDL, and GAS muscles in the LT, IBA, and ET groups were lower than that in the SA group, with decrements of 31–33% (SOL), 34–39% (EDL), and 23–26% (GAS) (Figure 4D). A similar alternation pattern in FKBP12 protein expression was observed in the SOL and EDL muscles. Compared with that in the SA group, FKBP12 protein expression in the SOL and EDL muscles increased by 24–105% in the PRE and LT groups but decreased by 22–25% in the ET group. Compared with that in the LT group, FKBP12 protein expression decreased by 16–59% (SOL) and 30–43% (EDL) in the IBA, ET, and POST groups. However, compared with that in the SA group, FKBP12 protein expression in the GAS muscle declined by 19–38% in the PRE, LT, and ET groups (*P* < 0.01) (Figure 4E).

### Relative Protein Expression Involved in SERCA1

In the SOL muscle, compared with that in the SA and PRE groups, SERCA1 protein expression increased by 23–40% in the IBA and ET groups. During the torpor-arousal cycle, SERCA1 protein expression in the IBA and ET groups was 28 and 16% higher than that in the LT group, respectively. In the EDL muscle, compared with that in the SA and PRE groups, SERCA1 protein expression increased by 38–64% in the LT, IBA, and ET groups. Furthermore, compared to that in the LT group, protein expression showed a 13 and 15% elevation in the IBA and ET groups, respectively. In the GAS muscle, compared with that in the SA group, SERCA1 protein expression increased by 50–100% in the PRE, LT, IBA, ET, and POST groups (Figure 4F). SLN protein expression in the three muscles of the PRE, LT, IBA, and ET groups was higher than that in the SA group, and was 19–29% lower in the ET group than that in the IBA group (Figure 4G). Phospholamban (PLB) protein expression in the SOL (ET), EDL (LT), and GAS (all other groups) was higher than that in the SA group (Figure 4H). As shown in Figure 4I, the p-PLB/PLB ratio in the SOL muscle was higher in the IBA group than that in the SA, PRE, and LT groups, but showed a decrease in the LT group, then recovery in the IBA, ET, and POST groups in the EDL muscle. In the GAS muscle, compared with that in the SA group, the ratio was elevated in the IBA, ET, and POST groups, and was much higher in the ET group than that in the LT group (Figure 4I). β-adrenergic receptor2 (β-AR2) protein expression in the SOL showed no change in the LT, IBA, and ET groups compared with that in the SA group, but decreased in the POST group. Compared with that in the SA, PRE, LT, and IBA groups, β-AR2 protein expression declined in the EDL muscle in the ET group. In the GAS muscle, compared with that in the SA group, β-AR2 protein expression increased (38–42%) in the PRE, IBA, and ET groups. Expression was also much higher in the IBA and ET groups than that in the LT group (Figure 4J). A similar calmodulin kinase 2 (CaMK2) protein expression trend was found in the SOL and EDL, with higher levels in the PRE, LT, IBA, and ET groups than that in the SA group. In the GAS muscle, compared with that in the SA and PRE groups, CaMK2 protein expression decreased by 16–53% in the LT, IBA, ET, and POST groups (Figure 4K). Compared with that in the PRE and LT groups, the P-CaMK2/CaMK2 ratio in the SOL muscle increased. However, it decreased by 12–62% and 14–64% in the EDL and GAS muscles, respectively, in all groups compared with that in the SA group (*P* < 0.01) (Figure 4L).

### Relative Protein Expression Involved in Ca^2+^-Binding Proteins

In the SOL muscle, during the torpor-arousal cycle, CaM protein expression was 46 and 44% higher in the IBA and ET groups, respectively, than that in the LT group. Similar change trends in CaM protein expression were observed in the EDL and GAS muscles, with higher levels in all groups (EDL, 58–153%; GAS, 68–150%) compared with that in the SA group (Figure 4M). Similar changes in CSQ1 protein expression were observed in the three different skeletal muscles, with higher levels in all groups (SOL, 140–360%; EDL, 72–112%; GAS, 104–176%) compared with the SA group. In addition, compared with levels in the PRE, LT, IBA, and ET groups, CaM protein expression decreased by 26–50% (SOL) and 53–62% (EDL) in the POST group (*P* < 0.01) (Figure 4N).

### Co-localization of Regulatory Proteins Involved in RyR1

**FIGURE 5:**
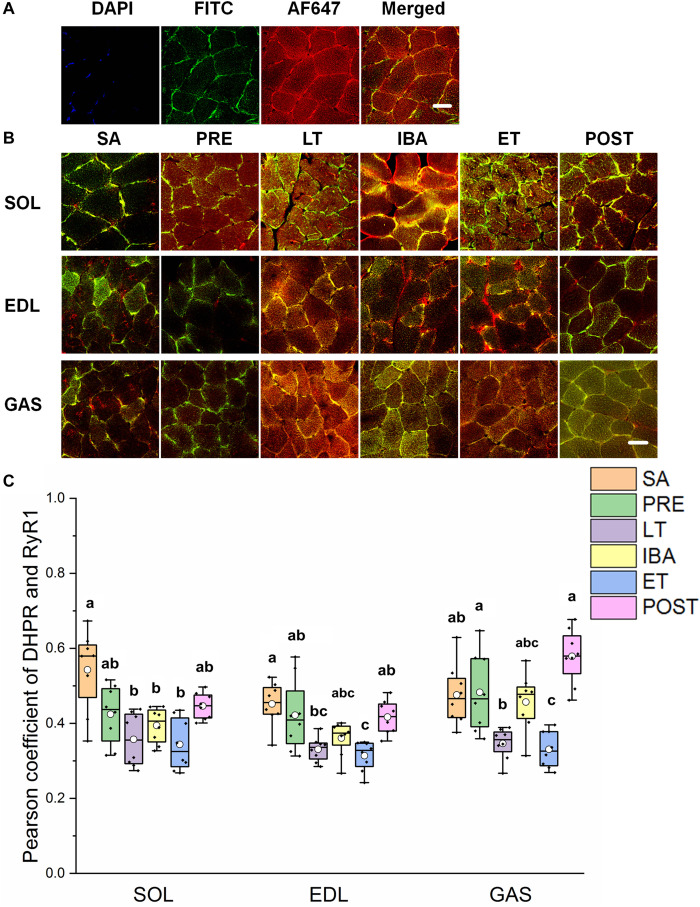
Protein overlap levels of DHPR and RyR1 in skeletal muscles during different periods. **(A)** Reticulate subcellular distribution of DHPR and RyR1 fluorescently labeled proteins in skeletal muscles. DAPI (blue) for nuclei, FITC (green) for DHPR, 647 (red) for RyR1. Scale bar = 20 μm. **(B)** Representative immunofluorescence of protein overlap between DHPR and RyR1 in three different types of muscle during different periods. Scale bar = 20 μm. **(C)** Box-plot representing protein overlap level of DHPR and RyR1. Boxes represent upper and lower quartiles, middle horizontal line represents median, hollow circle represents average, lines extending from upper and lower ends represent upper and lower edges, respectively, asterisks represent extreme outliers, and points represent individual sample values. *n* = 8. SOL, soleus muscle; EDL, extensor digitorum longus; GAS, gastrocnemius muscle; SA, summer active group; PRE, pre-hibernation group; LT, late torpor group; IBA, inter-bout arousal group; ET, early torpor group; POST, post-hibernation group. Different letters (such as a and b) indicate differences between period groups (*P* < 0.01), same letters (including a and ab) indicate no differences between period groups, and no letters indicate no differences among all six period groups.

**FIGURE 6:**
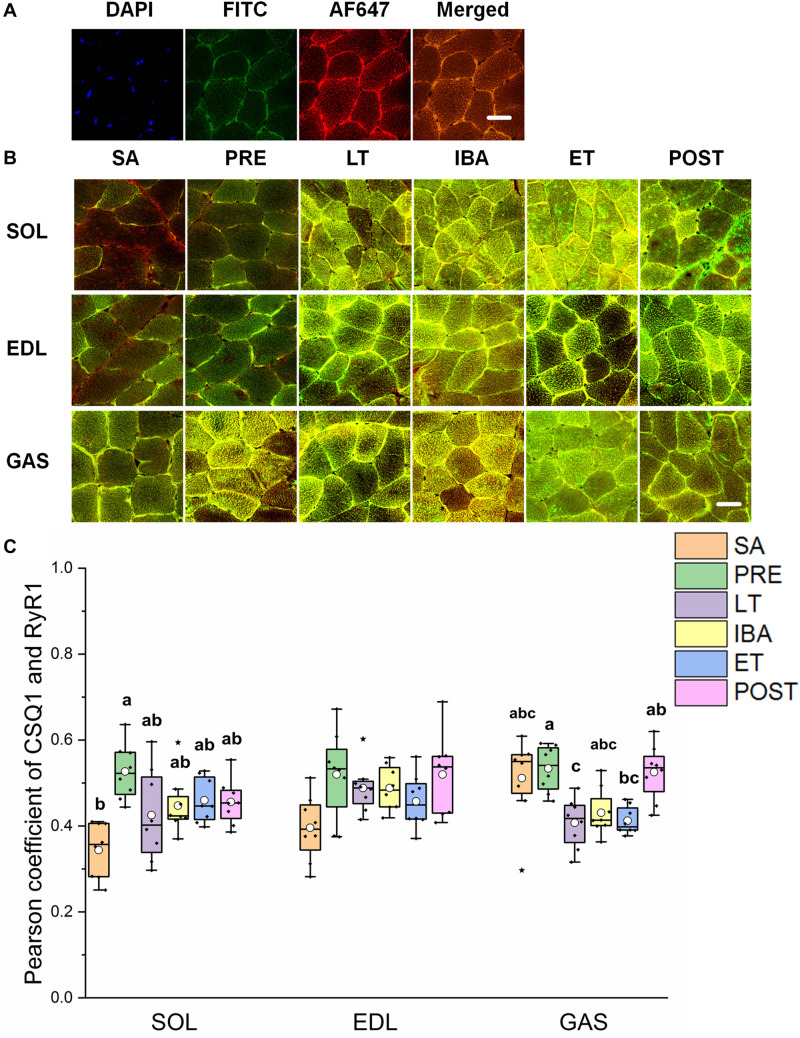
Protein overlap levels of CSQ1 and RyR1 in three different types of muscle during different periods. **(A)** Reticulate subcellular distribution of CSQ1 and RyR1 fluorescently labeled proteins in three different types of muscle. DAPI (blue) for nuclei, AF488 (green) for CSQ1, 647 (red) for RyR1. Scale bar = 20 μm. **(B)** Representative immunofluorescence of protein overlap between CSQ1 and RyR1 in three different types of muscle during different periods. Scale bar = 20 μm. **(C)** Box-plot representing protein overlap level of CSQ1 and RyR1. Boxes represent upper and lower quartiles, middle horizontal line represents median, hollow circle represents average, lines extending from upper and lower ends represent upper and lower edges, respectively, asterisks represent extreme outliers, and points represent individual sample values. *n* = 8. SOL, soleus muscle; EDL, extensor digitorum longus; GAS, gastrocnemius muscle; SA, summer active group; PRE, pre-hibernation group; LT, late torpor group; IBA, inter-bout arousal group; ET, early torpor group; POST, post-hibernation group. Different letters (such as a and b) indicate differences between period groups (*P* < 0.01), same letters (including a and ab) indicate no differences between period groups, and no letters indicate no differences among all six period groups.

As shown in Figures 5A,B, the co-localization levels of DHPR and RyR1 in the LT and ET groups were lower than that in the SA group in all three distinct skeletal muscles (Figure 5C). As shown in Figure 6, the co-localization levels of CSQ1 and RyR1 in the SOL muscle increased by 53% in the PRE group compared with that in the SA group. No change was found in the EDL muscle among all groups. In the GAS muscle, compared with that in the PRE group, levels decreased by 24 and 23% in the LT and ET groups, respectively (*P* < 0.01). The co-localization of RyR1 and FKBP12 was similarly measured and found to be unchanged among the six groups in all three skeletal muscles.

### Co-localization of Regulatory Proteins Involved in SERCA1

**FIGURE 7:**
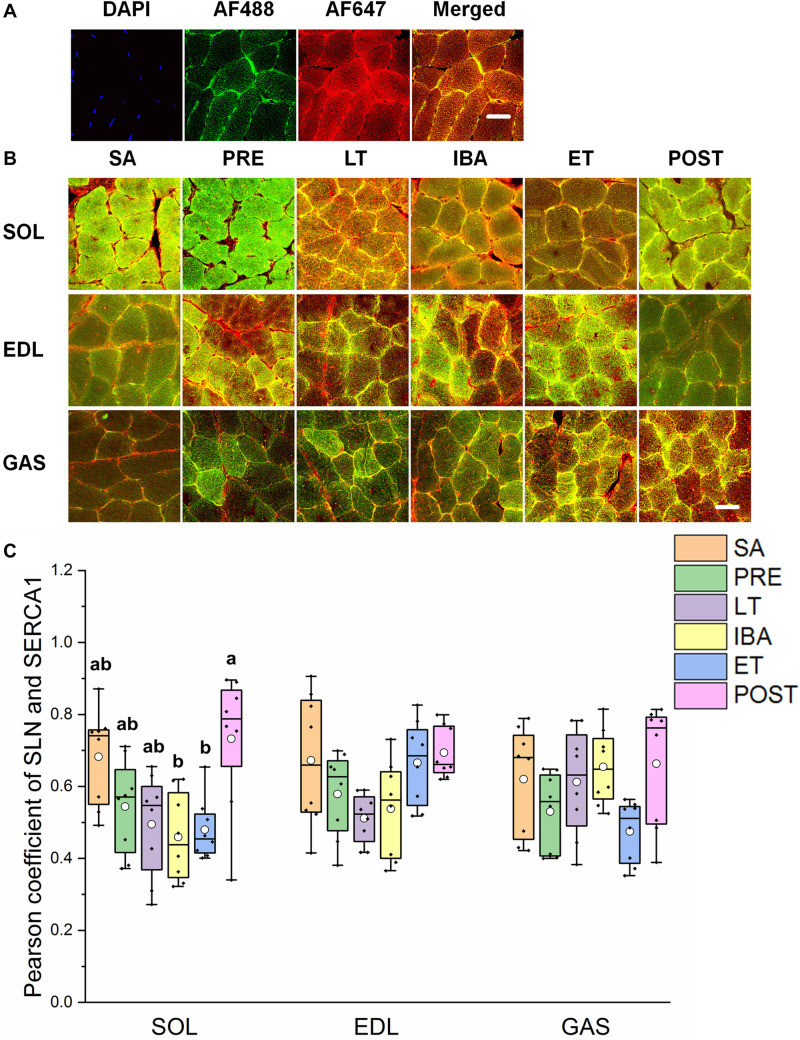
Protein overlap levels of SLN and SERCA1 in three different types of muscle during different periods. **(A)** Reticulate subcellular distribution of SLN and SERCA1 fluorescently labeled proteins in three different types of muscle. DAPI (blue) for nuclei, AF488 (green) for SLN, 647 (red) for SERCA1. Scale bar = 20 μm. **(B)** Representative immunofluorescence of protein overlap between SERCA1 and SLN in three different types of muscle during different periods. Scale bar = 20 μm. **(C)** Box-plot representing protein overlap level of SERCA1 and SLN. Boxes represent upper and lower quartiles, middle horizontal line represents median, hollow circle represents average, lines extending from upper and lower ends represent upper and lower edges, respectively, asterisks represent extreme outliers, and points represent individual sample values. *n* = 8. SOL, soleus muscle; EDL, extensor digitorum longus; GAS, gastrocnemius muscle; SA, summer active group; PRE, pre-hibernation group; LT, late torpor group; IBA, inter-bout arousal group; ET, early torpor group; POST, post-hibernation group. Different letters (such as a and b) indicate differences between period groups (*P* < 0.01), same letters (including a and ab) indicate no differences between period groups, and no letters indicate no differences among all six period groups.

The reticulate subcellular distributions of SLN and SERCA1 fluorescently labeled proteins are shown in Figures 7A,B. As seen in Figure 7C, compared with that in the SA and PRE groups, there was no change in the co-localization levels of SLN and SERCA1 in the three distinct skeletal muscles among the six groups; in the SOL muscle, however, levels were elevated in the POST group compared with that in the IBA and ET groups (*P* < 0.01) (Figure 7C).

## Discussion

An elevation in cytoplasmic Ca^2+^ concentration and a reduction in SR Ca^2+^ concentration were observed in the different skeletal muscles of the LT group compared to that of the SA group. This is similar to that found in non-hibernators under the disuse state (Ingalls et al., 2001; Hu et al., 2017), and indicates a degenerative change and loss of intracellular Ca^2+^ homeostasis. The elevation in Ca^2+^ concentration in all three skeletal muscles during late torpor was similar to the increase in cytoplasmic Ca^2+^ detected in the SOL of hibernating European hamsters (*Cricetus cricetus*) (Agostini et al., 1991). However, both Ca^2+^ concentrations recovered partially or completely in the ET group, thereby indicating the recovery of intracellular Ca^2+^ homeostasis. In addition, the changes in the cytoplasmic Ca^2+^ level were the opposite to those observed for SR Ca^2+^ during the “late torpor-inter-bout arousal-early torpor” cycle, indicating that the flow of Ca^2+^ between the cytoplasm and SR in skeletal muscle fibers was very active during the different stages in hibernating ground squirrels.

RyR1 protein expression increased in all three different skeletal muscles in the LT, IBA, and ET groups compared to levels in the SA and PRE groups, thus suggesting that SR Ca^2+^ release may have increased. The co-localization levels of DHPR and RyR1 in the three skeletal muscles decreased in the LT and ET groups compared to that in the SA and PRE groups. The structural combination of these two proteins is the basis for excitation-contraction coupling during skeletal muscle depolarization (Laver, 2018), and implies that the contraction function of skeletal muscle was weakened at this time, consistent with the fact that skeletal muscles are basically inactive during torpor. The co-localization levels of CSQ1 and RyR1 decreased, which may have increased the possibility of RyR1 channel opening to some extent, as CSQ1 is a negative regulator of RyR1 (Beard et al., 2002; Lee and Keener, 2008). Further, the co-localization levels of FKBP12 and RyR1 did not change in the three skeletal muscles in any of the six ground squirrel groups, suggesting that the inhibition of FKBP12 on RyR1 was stable during these periods.

Unexpectedly, we found that SERCA1 protein expression increased in the different skeletal muscles in the LT, IBA, and ET groups. We previously found that SERCA activity in the skeletal muscles of ground squirrels is up-regulated during torpor (Guo et al., 2017). This up-regulation of SERCA1 protein expression or activity in skeletal muscles indicates that the potential ability of SR Ca^2+^ uptake increased in these periods. In contrast, earlier research found that SERCA activity decreases in the hindlimb skeletal muscles of long-tailed ground squirrels (*Spermophilus undulatus*) during torpor (Malysheva et al., 2001), contrary to our findings on Daurian ground squirrels. These different results may be due to the different living environments of these two species. Long-tailed ground squirrels are mostly distributed in Siberia where the latitude is higher, winter is longer and colder, and the living environment is harsher. Therefore, the lower level of Ca^2+^ dynamic balance could reduce energy consumption and may be more conducive to survival. Phosphorylation of PLB, a key negative regulatory protein of SERCA, may reduce the inhibition effect of PLB on SERCA, resulting in the up-regulation of SERCA activity. The phosphorylation levels of PLB in the three different types of skeletal muscle (SOL in ET, EDL in PRE and LT, GAS in PRE, LT, IBA, ET, and POST) were increased, which may reduce the inhibition effect of PLB on SERCA (Asahi et al., 2003; Nicolaou et al., 2009; Shaikh et al., 2016). We found that β-AR2 protein expression in the GAS muscle increased in the other five groups compared with that in the SA group, which may explain the increased phosphorylation level of PLB (Hagemann and Xiao, 2002). SLN is another key negative regulatory protein of SERCA (Asahi et al., 2002; Akin et al., 2010). Our results showed that the co-localization levels of SLN and SERCA1 did not change in the three skeletal muscles in any group, suggesting that SLN might not be involved in the regulation of SERCA activity during these periods. Calmodulin kinase 2 (CaMK2) can enhance SERCA activity through autophosphorylation (Narayanan and Xu, 1997; Vangheluwe et al., 2005). Here, results showed that the phosphorylation level of CaMK2 increased in the SOL muscle but remained unchanged/decreased in the GAS and EDL muscles in the IBA group. This suggests that the increased phosphorylation level of CaMK2 may be an upstream signaling factor for increased SERCA activity in the slow-twitch SOL muscle of the IBA group. Earlier studies have suggested that the composition of polyunsaturated fatty acids (PUFA) in the myocardial membrane of Syrian hamsters (*Mesocricetus auratus*) and dormice (*Dryomys nitedula*) participates in the regulation of SERCA activity (Giroud et al., 2013, 2018). We speculated that the up-regulation of SERCA activity in the skeletal muscle of hibernating animals may also be associated with the components of the sarcoplasmic membrane; however, this needs further study.

It should be noted that RyR1 and SERCA1 were up-regulated in all three skeletal muscles in the ET, LT, and IBA groups. This could not fully explain why the cytoplasmic Ca^2+^ level increased in the LT stage but decreased in the IBA and ET stages. This contradiction suggests that regulation of the RyR1 channel may mediate the rate of SR Ca^2+^ release, and that regulation of SERCA-mediated Ca^2+^ uptake may be another potential mechanism leading to cytoplasmic Ca^2+^ increase during certain periods of hibernation, rather than during all stages of hibernation. In addition, from the perspective of energetics (Lo Martire et al., 2018; Silvani et al., 2018), the increased cytoplasmic Ca^2+^ level in the LT group may represent an increase in futile Ca^2+^ cycling, while it is necessary to increase energy expenditure to restore Ca^2+^ homeostasis through the SERCA-RyR Ca^2+^ cycle. This may prompt the animals to arouse from torpor. In other words, the increase in cytoplasmic Ca^2+^ during LT may be one of the factors resulting in inter-bout arousal following prolonged torpor (Grabek et al., 2019). Conversely, the reduction in cytoplasmic Ca^2+^ during IBA and ET could represent a reduction in futile cycling meant to reduce energy expenditure. Therefore, further relevant experiments, including SR function and energetics, are required to explore the mechanisms involved in this interesting phenomenon.

Cytoplasmic Ca^2+^ is also involved in intracellular Ca^2+^-binding proteins (Royer and Rios, 2009; Mekahli et al., 2011). Results showed that, compared with the SA group, the protein expression of CaM and CSQ1 in the different types of skeletal muscle increased by differing degrees, which would be beneficial for the decrease in cytoplasmic and SR free Ca^2+^ levels. This is similar to results reported in thirteen-lined ground squirrels, which show increases in protein expression in the SOL and GAS muscles during torpor (Zhang and Storey, 2016a, b).

## Conclusion

**FIGURE 8:**
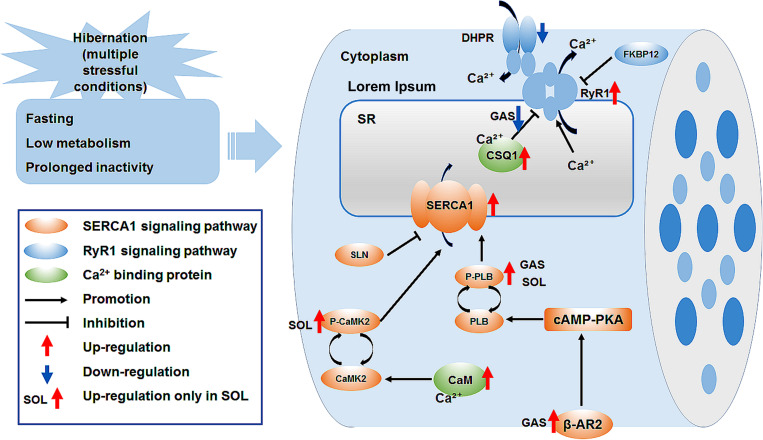
Graphical summary of study. Ca^2+^, calcium; SR, sarcoplasmic reticulum; DHPR, dihydropyridine receptor; FKBP12, 12kDa FK506 binding protein; RyR1, ryanodine receptor1; CSQ1, calsequestrin1; SERCA1, sarcoplasmic reticulum calcium ATPase; SLN, sarcolipin; PLB, phospholamban; P-PLB, phosphorylated phospholamban; CaM, calmodulin; CaMK2, calmodulin kinase2; P-CaMK2, phosphorylated calmodulin kinase2; β-AR2, β-adrenergic receptor 2; cAMP-PKA, cyclic adenosine monophosphate -protein kinase A signaling pathway.

In summary, the cytoplasmic Ca^2+^ level in skeletal muscle fiber increased, whereas the SR Ca^2+^ level decreased during late torpor. After inter-bout arousal and re-entry into torpor, the Ca^2+^ level recovered to varying degrees, indicating that intracellular Ca^2+^ is dynamic and the torpor-arousal cycle is very important for the recovery of intracellular Ca^2+^ homeostasis in the skeletal muscle of Daurian ground squirrels. The protein expression of RyR1 in skeletal muscles increased in the LT, IBA, and ET groups, suggesting that RyR1-mediated SR Ca^2+^ release may be elevated. The increase in SERCA1 and β-AR2 protein expression levels and P-PLB/PLB and P-CaMK2/CaMK2 ratios may have impacted the increase in SERCA-mediated SR Ca^2+^ uptake in the skeletal muscles of the IBA or ET groups. The high protein expression of Ca^2+^-binding CaM and CSQ1 enhanced the free Ca^2+^-binding capacity of skeletal muscle in all five groups. These results suggest that the high expression of RyR1 and increased probability of RyR1 channel opening may be potential mechanisms of SR Ca^2+^ release in the cytoplasm, resulting in an increase in cytoplasmic Ca^2+^ level; while the enhanced protein expression level of SERCA1 and high expression of the two free Ca^2+^-binding proteins may be possible mechanisms involved in the alleviation of the increase in cytoplasmic Ca^2+^ level and restoration of intracellular Ca^2+^ homeostasis (Figure 8).

## Limitations

In this study, the protein expression levels of RyR and SERCA and their co-localization with regulatory factors only reflected the potential biological functions of the Ca^2+^ channel or pump and their possible role in cytoplasmic Ca^2+^ changes during the different stages in hibernating ground squirrels. The regulation of RyR channel-mediated SR Ca^2+^ release and SERCA-mediated Ca^2+^ uptake may be the potential mechanisms involved in the increase in Ca^2+^ observed during certain periods of hibernation, rather than in all stages of hibernation. Therefore, the biggest drawback of this study is the lack of functional RyR and SERCA data (SR Ca^2+^ release and uptake). In addition to several Ca^2+^-handling-related proteins, cytoplasmic Ca^2+^ homeostasis is also affected by other Ca^2+^ stores (such as mitochondria) or Ca^2+^-binding proteins (such as parvalbumin). However, as the remaining skeletal muscle samples could not satisfy the needs of these experiments, the effects of these factors on cytoplasmic Ca^2+^ homeostasis during hibernation will be explored in future work.

## Data Availability Statement

All datasets presented in this study are included in the article/Supplementary Material.

## Ethics Statement

Ethics approval and consent to participate all animal procedures and care and handling protocols were approved by the Committee on the Ethics of Animal Experiments of the Northwest University (Permit Number: SYXK 2010-004).

## Author Contributions

Y-FG, ZW, and H-PW conceived and designed the experiments. ZW, X-FM, JZ, HC, XP, and S-HX performed the experiments. ZW and X-FM analyzed the data. ZW, JZ, and Y-FG wrote the manuscript. All authors contributed to the article and approved the submitted version.

## Conflict of Interest

The authors declare that the research was conducted in the absence of any commercial or financial relationships that could be construed as a potential conflict of interest.

## Abbreviations

CaM: calmodulin
CaMK2: calmodulin kinase 2
Ca^2+^: calcium
CSQ1: calsequestrin1
DAPI: 4 ′ -6 ′ -diamidino-2-phenylindole
DHPR: dihydropyridine receptor
EDL: extensor digitorum longus
ET: early torpor
FKBP12: 12-kDa FK506 binding protein
Fluo-3/AM: fluo-3-acetoxymethylester
IBA: inter-bout arousal
GAS: gastrocnemius
LT: late torpor
mag-Fluo-4/AM: magnesium-Fluo-4-acetoxymethylester
MWW: skeletal muscle wet weight
MWW/BW: ratio of skeletal muscle wet weight to body weight
PLB: phospholamban
POST: post-hibernation
PRE: pre-hibernation
RT-PCR: real-time PCR
RyR1: ryanodine receptor1
SA: summer active
SERCA1: sarco/endoplasmic reticulum Ca^2+^ ATPase isoform 1
SLN: sarcolipin
SOL: Soleus
SR: sarcoplasmic reticulum
β-AR 2: β-adrenergic receptor2.
