# Supplementary material for: A Temporal Examination of Cytoplasmic Ca2 + Levels, Sarcoplasmic Reticulum Ca2 + Levels, and Ca2 + -Handling-Related Proteins in Different Skeletal Muscles of Hibernating Daurian Ground Squirrels
Source: Front Physiol. 2020 Oct 21;11:562080. doi: 10.3389/fphys.2020.562080 (PMC7609816; doi:10.3389/fphys.2020.562080)
Supplement: Supplementary file 1 [file Data_Sheet_1.PDF]

Original figures of western blot

Original figures of RYR1

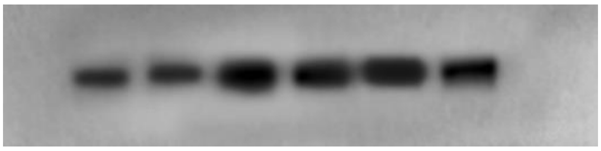

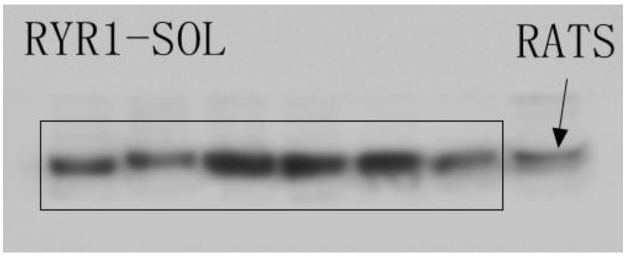

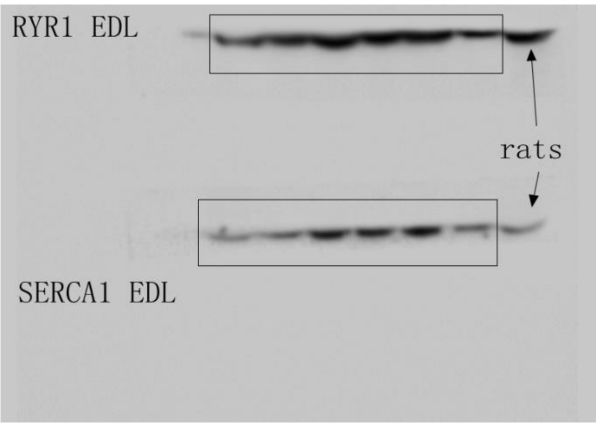

Original figures of SERCA1

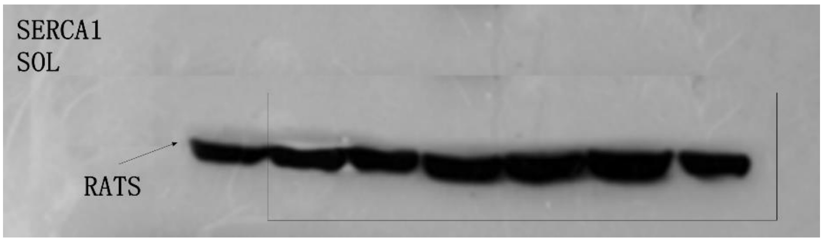

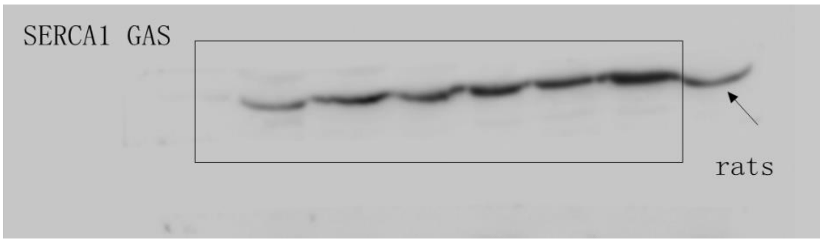

Original figures of DHPR

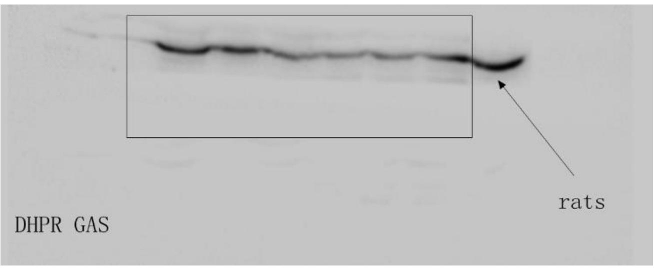

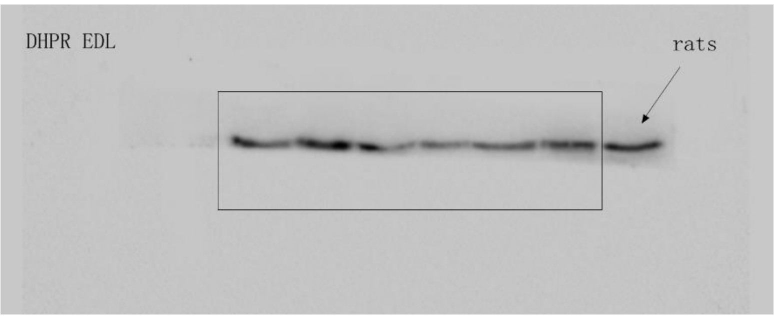

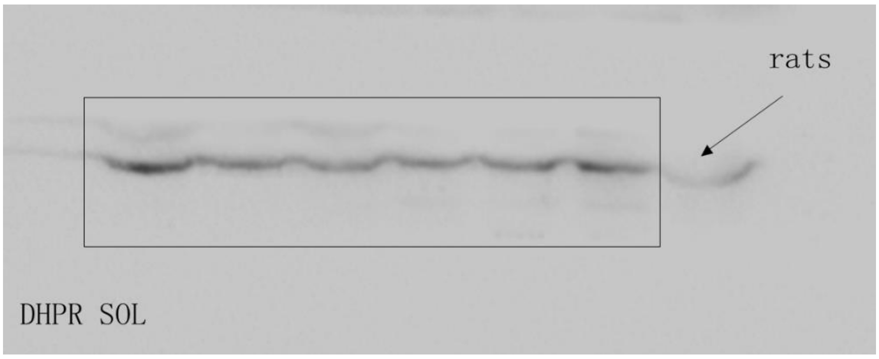

Original figures of FKBP12

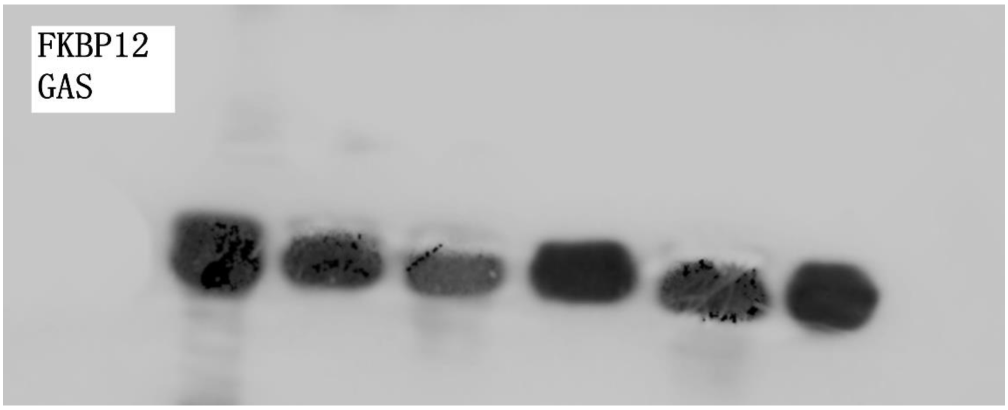

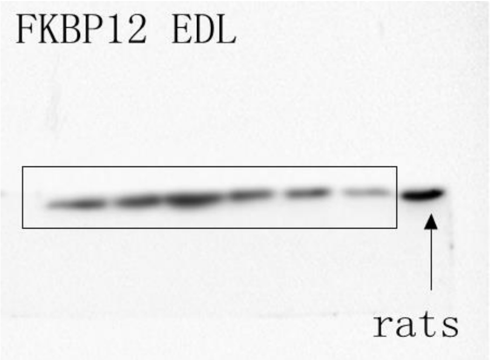

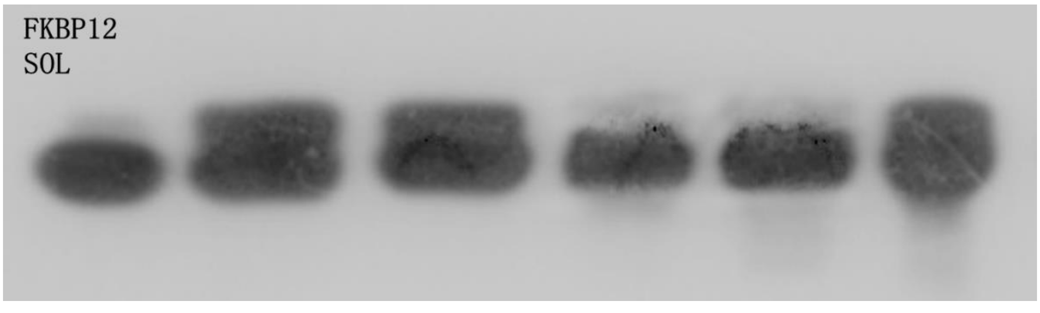

Original figures of SLN

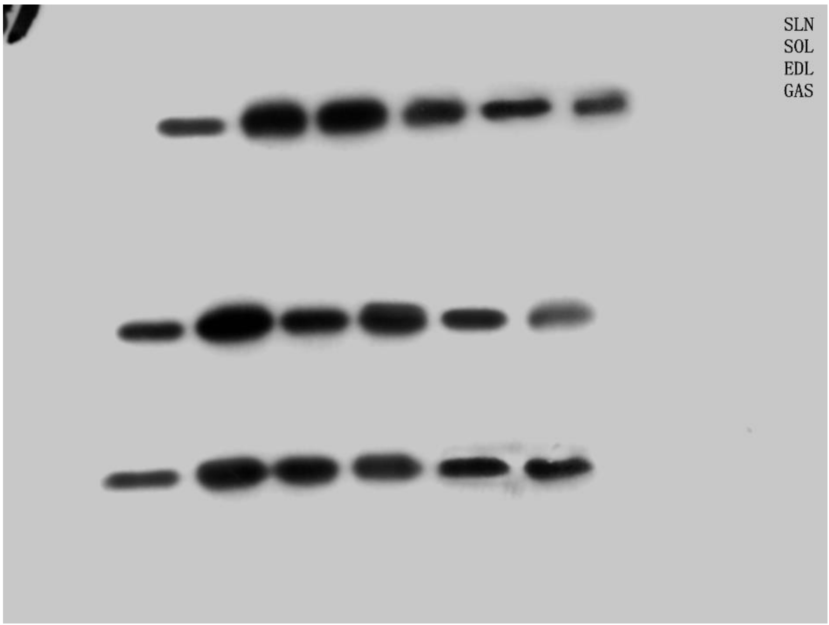

Original figures of PLB

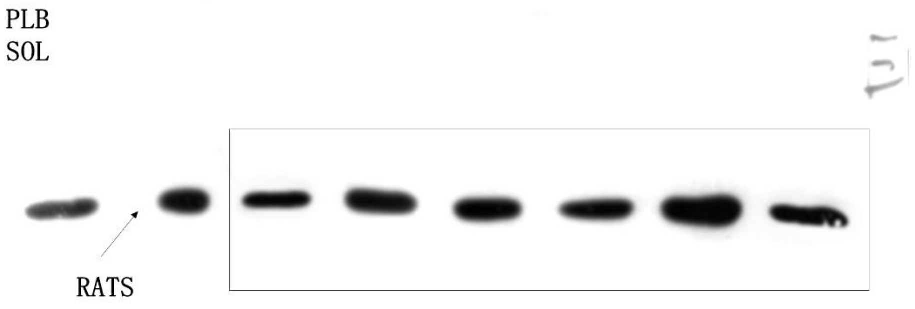

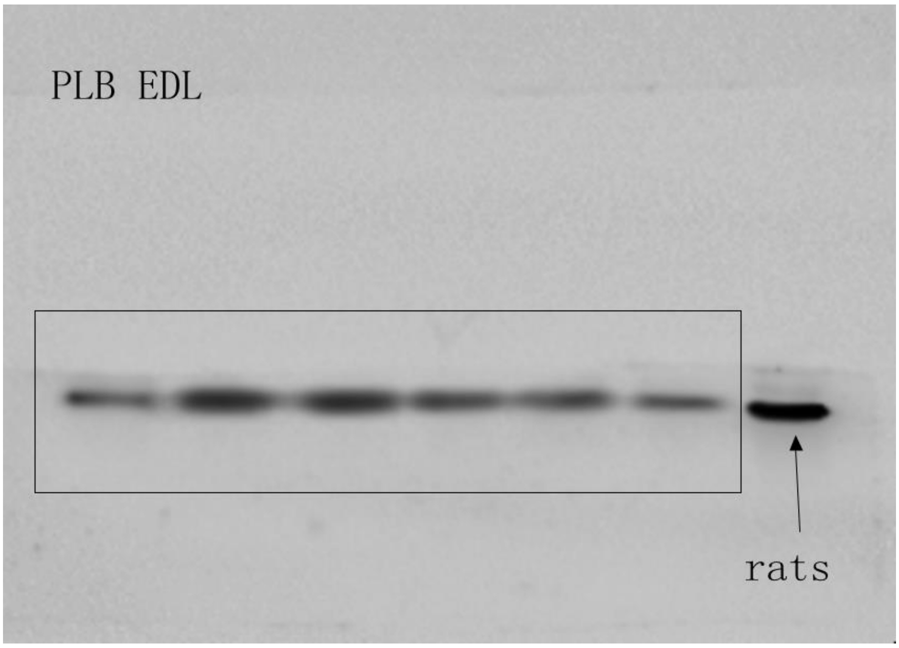

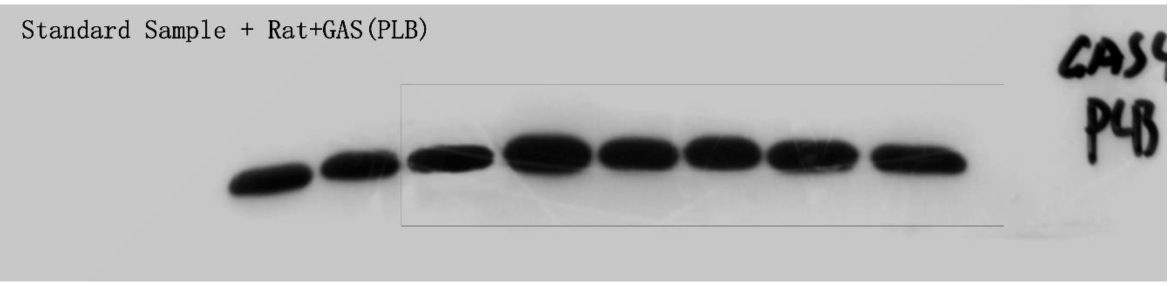

Original figures of P-PLB

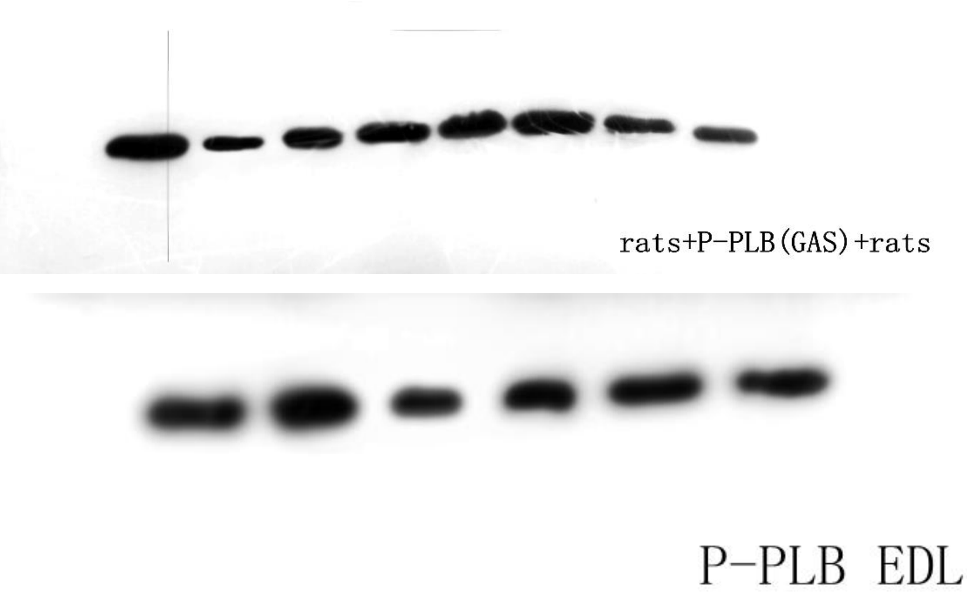

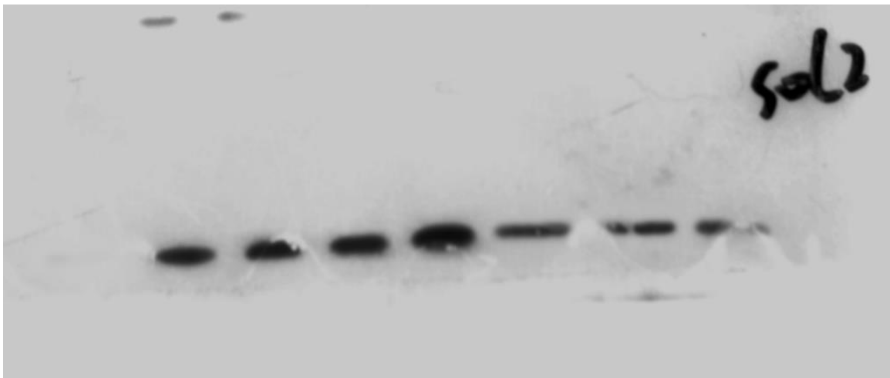

Original figures of beta-AR2

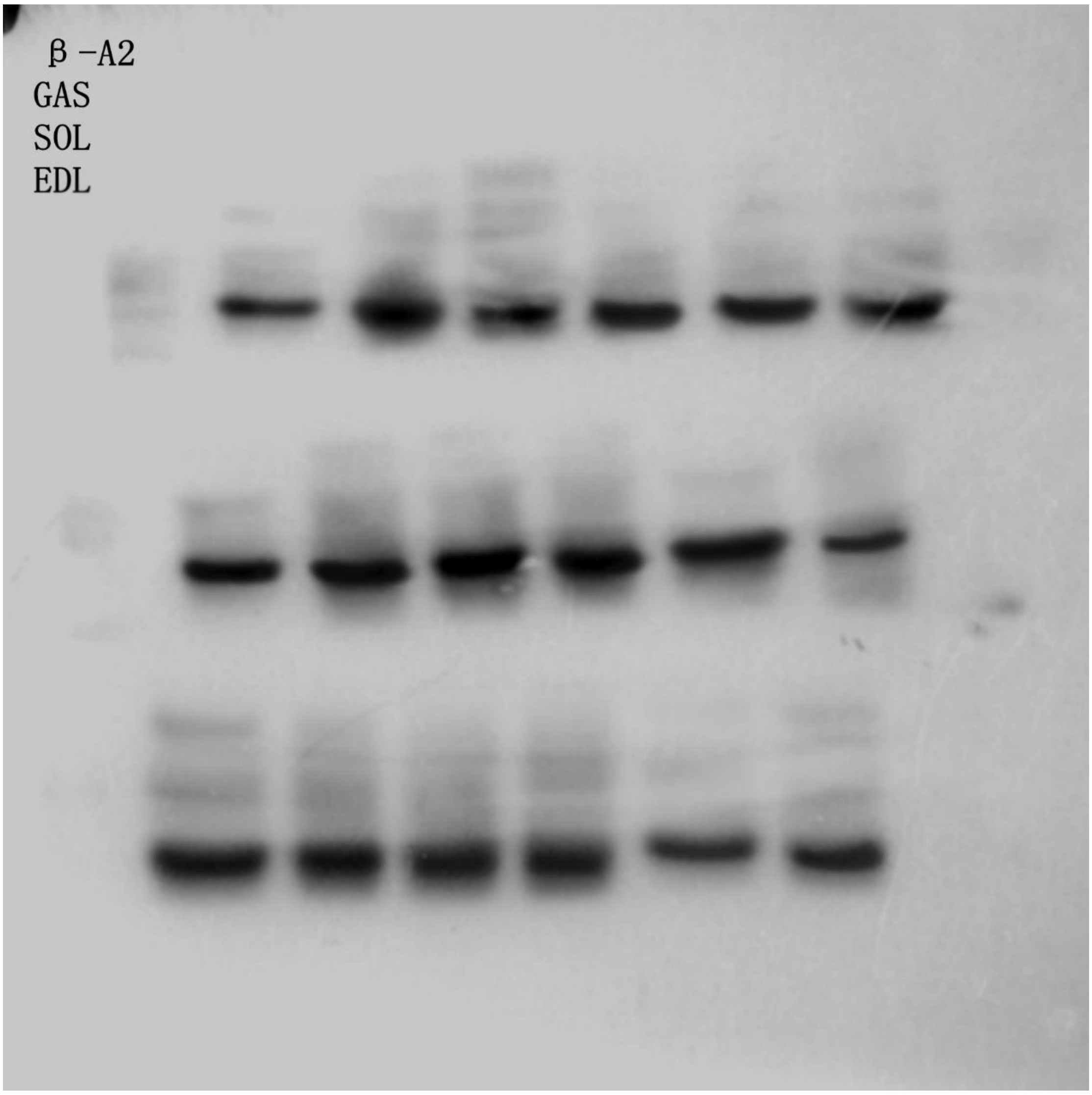

Original figures of CAMK2

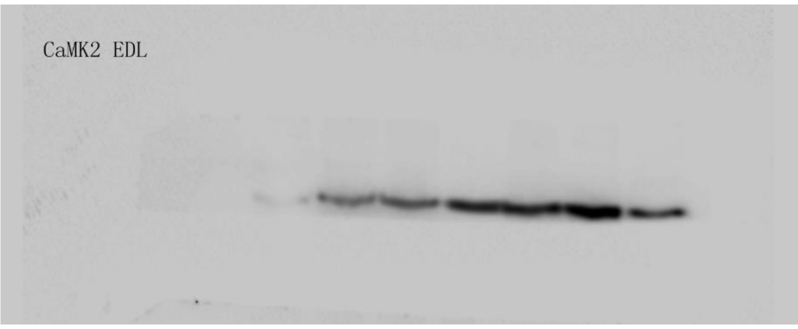

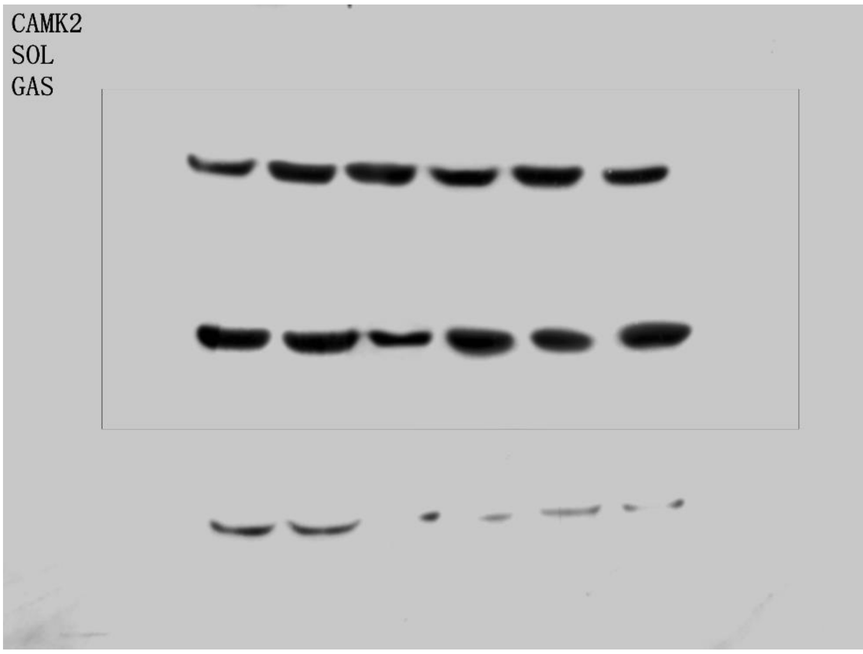

Original figures of P-CAMK2

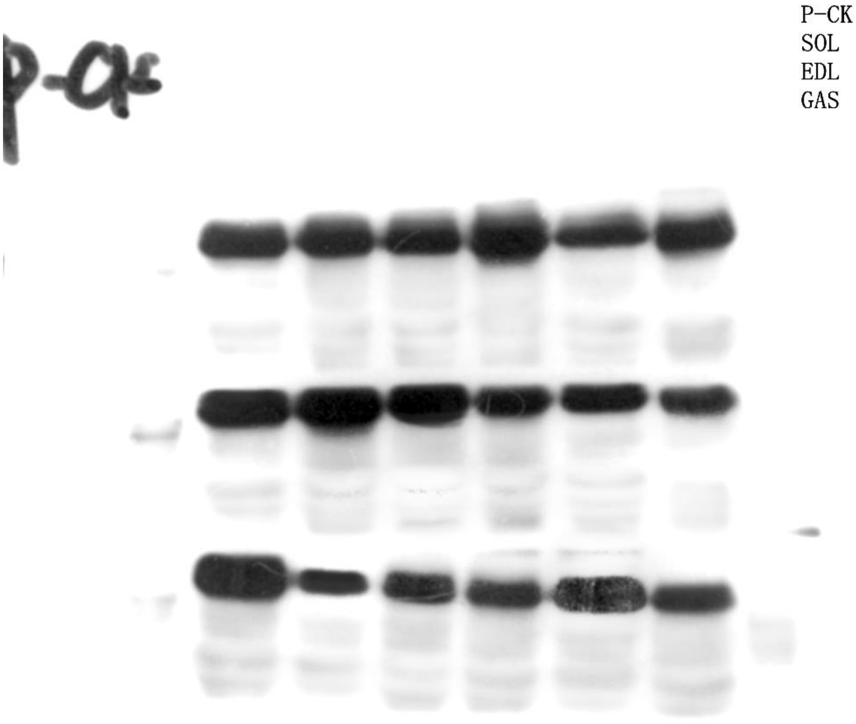

Original figures of CaM

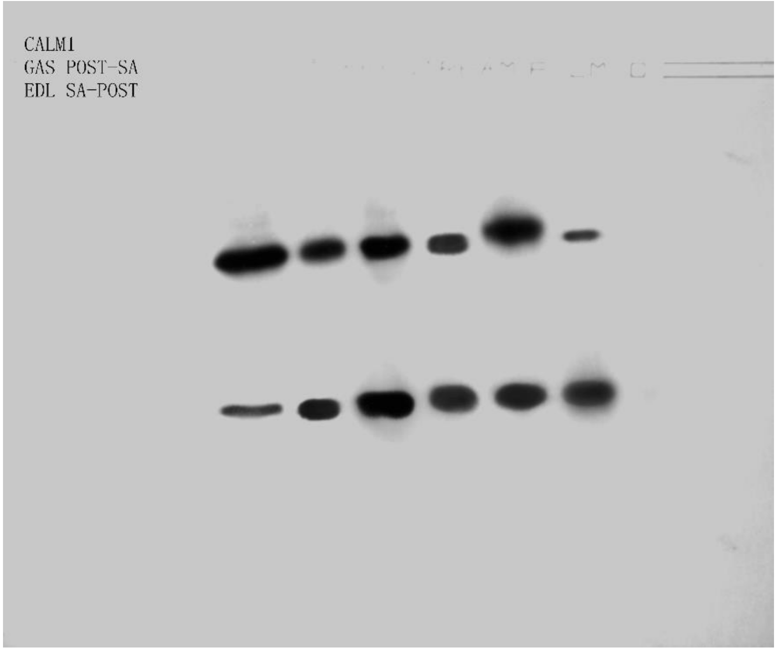

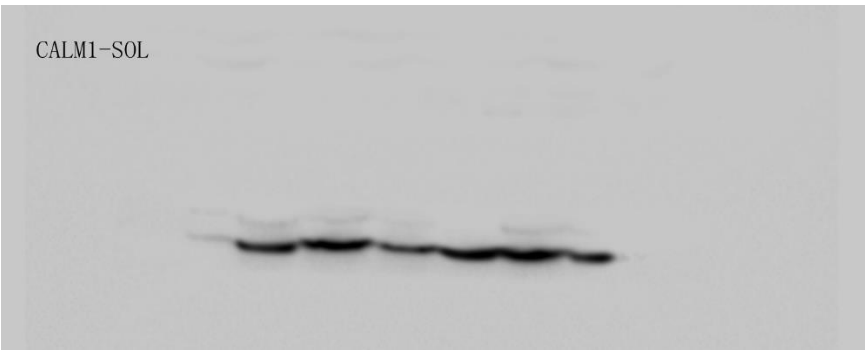

Original figures of CSQ1

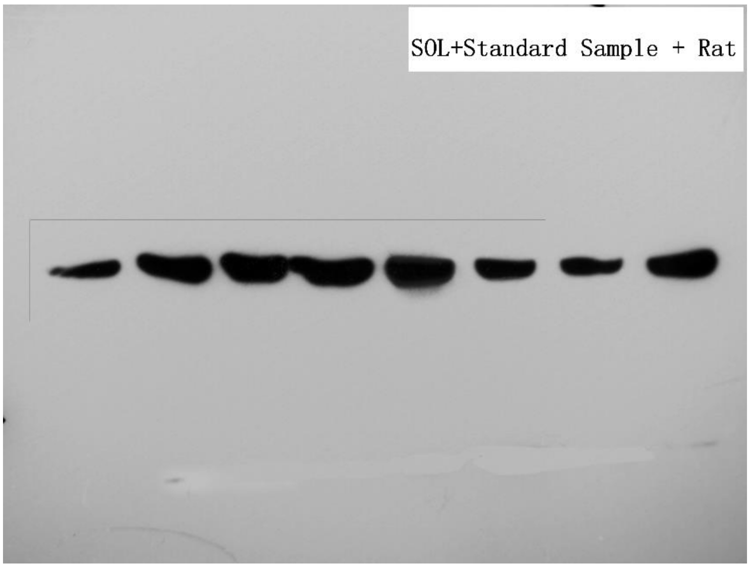

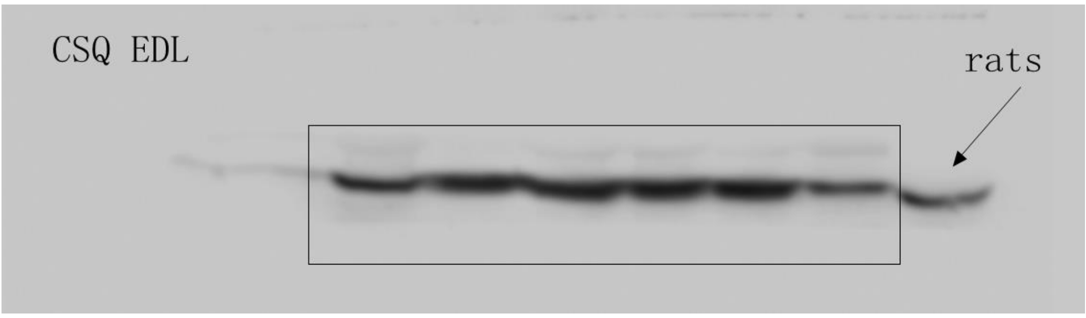
CSQ EDL
rats

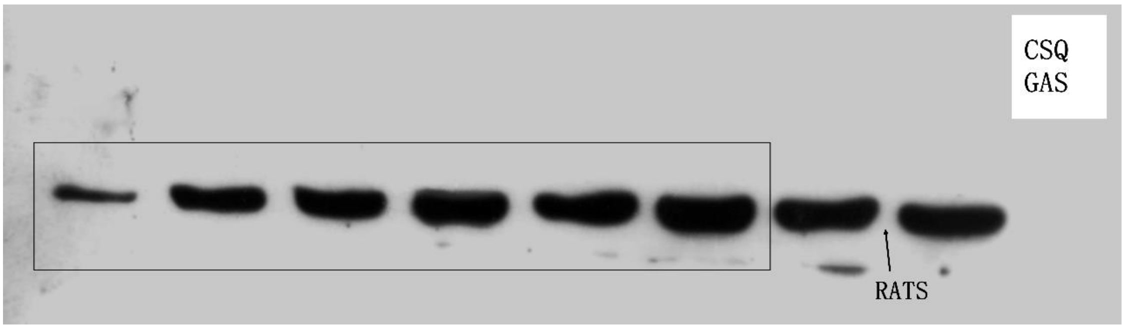
CSQ
GAS
RATS
